# Validation of the biological function and prognostic significance of AURKA in neuroblastoma

**DOI:** 10.1371/journal.pone.0313939

**Published:** 2024-11-25

**Authors:** Jing Chu

**Affiliations:** Department of Pathology, Anhui Provincial Children’s Hospital, Hefei, Anhui, China; Centre de Recherche en Biologie cellulaire de Montpellier, FRANCE

## Abstract

**Background:**

Neuroblastoma (NB) is the most common extracranial solid tumor in children, and the AURKA gene encodes a protein kinase involved in cell cycle regulation that plays an oncogenic role in a variety of human cancers. The aim of this study was to validate the biological function and prognostic significance of AURKA in NB using basic experiments and bioinformatics.

**Methods:**

Data obtained from Target and GEO databases were analyzed using various bioinformatic techniques. The expression of AURKA in 77 NB samples was detected by immunohistochemistry (IHC) method. The lentiviral RNA interference technique was employed to downregulate AURKA gene expression in NB cell lines. Additionally, cell counting kit-8 and flow cytometry analysis were conducted to investigate the impact of AURKA expression on cell proliferation, cell cycle progression, and apoptosis.

**Results:**

A bioinformatic analysis showed that patients with NB in the AURKA-high-expression group had shorter OS (Overall Survival). Immune cell infiltration analysis showed that only activated CD4 T cell and type 2 T helper cell infiltration levels were higher in the AURKA-high-expression group than in the AURKA-low-expression group, with the infiltration levels of most other immune cells and cytokines lower in the high-expression group. Furthermore, the enhanced infiltration of activated CD4 T cells was associated with worse OS in patients with NB.

IHC results showed that the AURKA expression was correlated with MYCN status and INSS stage. Log-rank test showed that pathological type, MYCN status, INSS stage, COG risk group, and AURKA expression was related to PFS (Progression-free survival) of NB patients, but COX regression analysis showed that none of the above factors were independently prognostic for PFS.

In vitro, shRNA delivered via an AURKA-specific lentivirus significantly and consistently silenced endogenous AURKA expression in the human NB cell line SK-N-AS. This inhibited tumor cell proliferation, induced apoptosis, and caused G2/M-phase cell cycle arrest. Moreover, western blot assay showed significant reductions in the levels of mTOR, p70S6K, and 4E-BP1 phosphorylation in the AURKA-knockdown group. I found in subsequent experiments that NFYB can bind to the AURKA promoter and thus promote AURKA expression.

**Conclusions:**

High-level AURKA expression in NB is associated with poor patient prognosis. Silencing AURKA inhibited tumor cell proliferation, induced tumor cell apoptosis, and led to cell cycle arrest in the G2/M phase. Mechanistically, AURKA knockdown inhibited the phosphorylation and the activation of the mTOR1/p70S6K/4E-BP1 signaling pathway. In addition, AURKA was observed to regulate the infiltration levels of various immune cells in the NB tumor microenvironment, resulting in remodeling of the immunosuppressive tumor microenvironment.

## Introduction

Neuroblastoma (NB) is the most common extracranial solid tumor in children, originating from primitive neuroepithelial cells of the embryonic neural crest, and it accounts for 15% of cancer-related deaths in children [1]. The presence of a MYCN gene amplification, occurring in approximately 20% of NB cases, is one of the strongest predictors of poorer overall survival (OS) in high-risk patients, and recurrence still occurs in half of high-risk patients after a combination of surgery, radiotherapy, high-dose chemotherapy, differentiation therapy, and GD2-targeting monoclonal antibody immunotherapy, with the 5-year survival rate after relapse being less than 10% [1].

AURKA, located on human chromosome 20q13, encodes a member of the serine-threonine kinase family that plays an important role in centrosome maturation and separation, spindle assembly, and cell cycle regulation [2]. The significance of AURKA as a therapeutic target in NB arises from its dual role, encompassing both catalytic functions during mitosis and kinase-independent functions, notably the stabilization of the crucial oncoprotein MYCN [3]. Elevated levels of AURKA expression have been associated with unfavorable overall and event-free survival outcomes in patients with NB, as evidenced by previous studies [4]. Treatment of NB cells with alisertib, a specific AURKA inhibitor, inhibited cell growth, degraded MYC protein levels, and resulted in inhibition of tumor growth in a xenograft mouse model [5]. However, the efficacy of small molecule inhibitors targeting AURKA in clinical trials has not met expectations, despite their promising potential. In clinical trials of AURKA inhibitors, the activity of alisertib was lower in MYCN-amplified NB [6]. AT9283 completed phase I without finding objective responses in three NB patients [7]. The underlying mechanism of AURKA in NB remains inadequately understood. In this study, AURKA expression, its pathological significance, and potential molecular mechanisms in NB using bioinformatics and molecular biological experimental methods were investigated.

## Materials and methods

### Data acquisition, group division and survival analysis

Processed raw mRNA expression data for NB were downloaded from the TARGET database(https://portal.gdc.cancer.gov/projects/TARGET-NBL), including data for 153 NB samples (S1 and S2 Files). A Series Matrix File of GSE49710 was downloaded from the NCBI GEO public database (https://www.ncbi.nlm.nih.gov/geo/query/acc.cgi?acc=GSE49710). Data for 498 patients with NB was extracted, which included expression profiles and survival information (S3 and S4 Files). The data were accessed for research purposes between July and December 2023.

Based on the AURKA expression and survival data from the TARGET and GSE49710 datasets, patients were categorized into AURKA-high-expression and low-expression groups after calculating the cutoff value with the maximum rank statistic [8]. The correlation between AURKA expression and the OS of patients with NB was evaluated using the R package “*survival*”, and survival curves were generated using the R package “*survminer*” [9]. Student’s t test [10] estimated the correlation between clinical pathological features and AURKA expression. The “ggplot2” packages was used for visualization.

### Correlation and enrichment analyses

Pearson correlation analysis [11] of AURKA mRNA and other mRNAs was performed in NB using TARGET and GEO data. Gene set enrichment analysis (GSEA) [12] was performed using the gseReactome functions in clusterProfiler [13]. The HALLMARK, KEGG, and Reactome pathways were downloaded from the Msigdb database [14], and the pathways were scored using the R package GSVA (gene set variation analysis) [15] to evaluate pathway differences between high and low AURKA expression. The R package “pheatmap” was used to generate heatmaps.

### Immune infiltration analysis

The differences in immune cell infiltration in the tumor microenvironment (TME) between the high and low AURKA expression groups were further analyzed to explore the immune-related mechanisms of AURKA in NB. The correlation between AURKA expression and the infiltration abundance of 23 immune cells in NB was assessed using the ssGSEA (single-sample Gene Set Enrichment Analysis) [16] function of the GSVA package in R and Pearson correlation analysis.

Next, patient samples with activated CD4 T cells were extracted from the two databases and sorted from low to high according to the proportion of these cells. Using the median value as a boundary, the samples were categorized into activated CD4 T cell-high and activated CD4 T cell-low groups. The downloaded clinical data were integrated and then analyzed using the “*survival”* package to perform proportional hazards hypothesis testing and fit survival regression. The results were visualized using the “*survminer”* and ggplot2 packages.

The "ESTIMATE" package [17] in R was used to analyze the correlation of stromal, immune, and ESTIMATE scores with AURKA expression. The TISIDB database [18] was used to analyze AURKA expression and the TME of NB, including chemokines and their receptors, interleukins and their receptors, interferons and their receptors, and other cytokines.

### Protein-protein interaction (PPI) and transcription factors (TFs)–miRNA—mRNA regulatory networks

Normal cellular activities depend on complex networks of functional associations among biomolecules. Within these networks, PPIs are particularly important because of their versatility, specificity, and adaptability. The STRING database(https://cn.string-db.org/) [19] is dedicated to integrating all known and predicted associations between proteins, including physical interactions and functional associations. The STRING database was used to construct a PPI network diagram of AURKA. The RegNetwork database(https://regnetworkweb.org/) [20], a comprehensive resource for human transcriptional and post-transcriptional regulatory networks, was employed to predict upstream miRNAs and TFs that interact with AURKA, facilitating the study of its regulatory mechanisms in NB. Network diagrams were created using Cytoscape software (v 3.7.2).

### Drug sensitivity analysis

The drug sensitivity of each tumor sample was predicted using the “pRRophetic” package of R [21]. Ridge regression was used to determine the half-maximal inhibitory concentration (IC_50_) for the samples. Moreover, Spearman correlation analysis was used to determine the correlation between the drug IC_50_ value and AURKA expression.

### Collection of clinical specimens and Immunohistochemistry (IHC)

The paraffin-embedded tissue samples of 77 patients with NB were obtained from July 1, 2012 to December 31, 2023 at Anhui Provincial Children’s Hospital with written informed consent of the patients or their guardians. The author had access to information that could identify individual participants during collection between July and December 2023. All procedures were performed in accordance with the principles of the Declaration of Helsinki and approved by the Anhui Provincial Children’s Hospital Medical Research Ethics Committee (EYLL-2022-034). The primary antibody used was AURKA (Abcam, ab52973, at 1/100 dilution). PBS was used to instead of primary antibody in the negative control. The evaluation of AURKA positivity by IHC was performed by the Barnes’ score [3].

### Cell culture

Human NB tumor cell line (SK-N-AS) was purchased from Wuhan Procell Life Technology Co., Ltd. (Hubei, China) and cultured in DMEM (Gibco, C11965500BT) supplemented with 10% FBS(Gibco, 10270–106), 100 U/ml penicillin and 100 μg/ml streptomycin at 37˚C under humidified air containing 5% CO_2_.

### AURKA gene silence mediated by lentivirus-delivered short hairpin RNA (shRNA)

AURKA-targeting short hairpin RNA (sh-AURKA, 5’-GCAGAGAACTGCTACTTAT AT-3’) and a nonspecific control (sh-NC, 5’-TTCTCCGAACGTGTCACGT-3’) were constructed using a pCLenti-U6-shRNA-CMV-Puro-WPRE lentiviral vector (OBiO Technology Co., Ltd., Shanghai, China). The recombinant virus was packaged using the Lentivector Expression systems (OBiO Technology). SK-N-AS cells were infected. Cells were selected for at least 14 days after two rounds of infection by adding 1 g/mL Puromycin (J593, Amresco) to growth medium.

### Reverse transcription-quantitative PCR (RT-qPCR)

The method was described in my previously published paper [22]. These RNAs were extracted by TRIzol reagent (Invitrogen) and reverse transcribed by HiScript III RT SuperMix for qPCR (Vazyme Biotech). The sequences of the primers were as follows: AURKA forward: 5’-ACCTGTTAAGGCT ACAGCTCCA-3’, AURKA reverse:5’-AAGGACACAAGACCCGCTGA-3’; nuclear transcription factor Y subunit beta (NFYB) forward: 5’-ACACTGTTCATCGTGCCTGT-3’, NFYB reverse: 5’-GGGCACCATTTCAAGAGCAC-3’; GAPDH forward: 5’-GTCTTCACCACCATG GAGAA-3’, GAPDH reverse:5’-TAAGCAGTTGGTGGTGCAG-3’. The mean and standard error for each point were calculated for each sample in three separate reactions. The relative levels of AURKA mRNA transcripts were normalized to the control GAPDH. Relative gene expression was quantified using the GraphPad Prism 5.0 software.

### Western blot

The cells were lyzed in a RIPA lysis buffer containing protease inhibitors (P0013, Beyotime Biotechnology, Shanghai, China). The concentration of protein was evaluated by using Pierce bicinchoninic acid (BCA) Protein Assay Kit (Thermo, 23225)according to the manufacture’s protocol. Western blot analysis was performed according to previous experimental procedures [23]. Primary antibodies included AURKA(1:1000 dilution, catalog ab52973, Abcam), phospho-mTOR(p-mTOR, Ser2448; 1:1000, #5536, Cell Signaling), total mTOR(1:1000, #2983, Cell Signaling), phosphor-p70S6K(p-p70S6K,Thr389; 1:1000, #9234, Cell Signaling), total p70S6K (1:1000, #9202, Cell Signaling), phospho-4E-BP1(p-4E-BP1,Thr37/46; 1:1000, 2855T, Cell Signaling), total 4E-BP1(1:2000, #9644, Cell Signaling), and NFYB (1:1000, ab111577, Abcam). An anti-GAPDH (1:1000, #2118, Cell Signaling) antibody was used to normalize the quantity of the loaded samples.

### Cell counting kit-8 assay

Cell proliferation was determined using a Cell Counting Kit-8 (CCK-8) kit (40203ES76, Yeasen Biotechnology Co., Ltd., Shanghai, China) according to the instruction provided by the producer. The experiment was repeated three times in triplicate for each assay.

### Cell cycle analysis

SK-N-AS cells from WT, sh-NC and sh-AURKA group, respectively were collected by centrifugation, washed thrice with cold PBS, and fixed with 70% ethanol at 4°C overnight. The fixed cells were resuspended in PBS containing 10μL of propidium iodide (PI, 50μg/mL) (C1052, Beyotime, Shanghai, China) and 5μL of 10 mg/mL RNase A, then incubated at 37°C for 30 min in a dark place. The analysis of cell cycle distribution was performed by a flow cytometer (NovoCyte, ACEA, California, USA) in accordance with the manufacturer’s guidelines. A minimum of three replicates were performed for each assay.

### Apoptosis assay

Annexin V-FITC/PI Apoptosis Detection Kit (C1062M, Beyotime, Shanghai, China) was employed to detect apoptosis. Flow cytometry (NovoCyte, ACEA, California, USA) was used to analyze the the proportion of apoptotic cells. An experiment was repeated at least three times for each assay.

### Dual-Luciferase assay

The pGL3 plasmid was utilized to build luciferase reporter plasmids containing mutant and wild-type AURKA promoter sequences (Promega, USA). Reporter plasmids were co-transfected with either oe-NC or oe-NFYB into SK-N-AS cells. Luciferase activity was assayed on Dual-Luciferase Reporter Gene Assay System (Promega, USA).

### Statistical analysis

All bioinformatics statistical analyses were performed using R language (version 4.2.2). All statistical tests were bilateral, and *P*<0.05 was considered statistically significant.

Data from IHC experiments were statistically analyzed using SPSS 26.0. Categorical data were expressed as the number of cases (percentage), and comparisons between groups were made using the χ2 test [10]. Logistic regression [24] was used to analyze the relevant factors affecting the expression of AURKA. Kaplan-Meier survival analysis and log-rank tests [24] were used to compare the PFS (Progression-free survival) of patients with different clinical characteristics. The COX proportional risk model [24] was used to analyze factors affecting patient disease progression or mortality. PFS was defined as the time from the diagnosis to tumor recurrence or death. Test results were considered statistically significant at *P*<0.05.

Data analysis from cytological experiments was performed using GraphPad 8.0 software. Measurement data are expressed as mean ± standard deviation. One-way ANOVA [25] was used to compare the means of three or four groups. Student’s t test was used to compare the means of two groups. *P*<0.05 was considered statistically significant.

## Results

S1 Fig shows the overall workflow diagram of this study.

### 1 Bioinformatic analysis results

#### 1.1 Relationship between AURKA expression and prognosis of patients with NB

**Fig 1:**
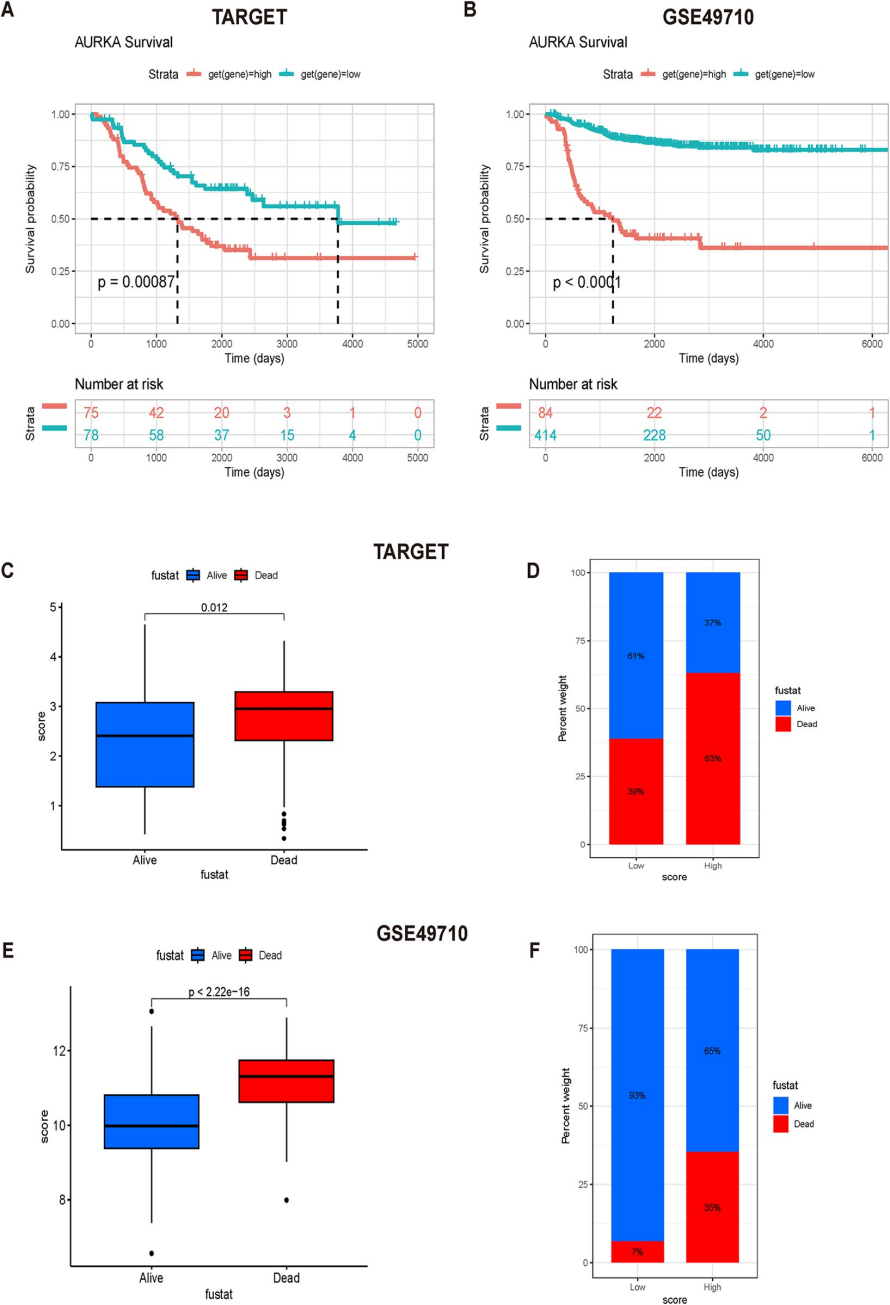
Relationship between AURKA expression and prognosis of patients with neuroblastoma. (A, B) High AURKA expression was negatively associated with overall survival in patients with neuroblastoma. (C-F) The level of AURKA expression was higher in patients who died, and the percentage of patients who died was higher in the AURKA-high-expression group.

Survival analysis using the TARGET database and the GSE49710 dataset showed that high AURKA expression was negatively associated with OS in patients with NB (Fig 1A and 1B). Moreover, Student’s t test showed that the level of AURKA expression was higher in patients who died, and the percentage of patients who died was higher in the AURKA-high-expression group (Fig 1C–1F). These results suggested that patients with NB in the AURKA-high-expression group had a poor prognosis.

#### 1.2 Correlation and enrichment analysis

**Fig 2:**
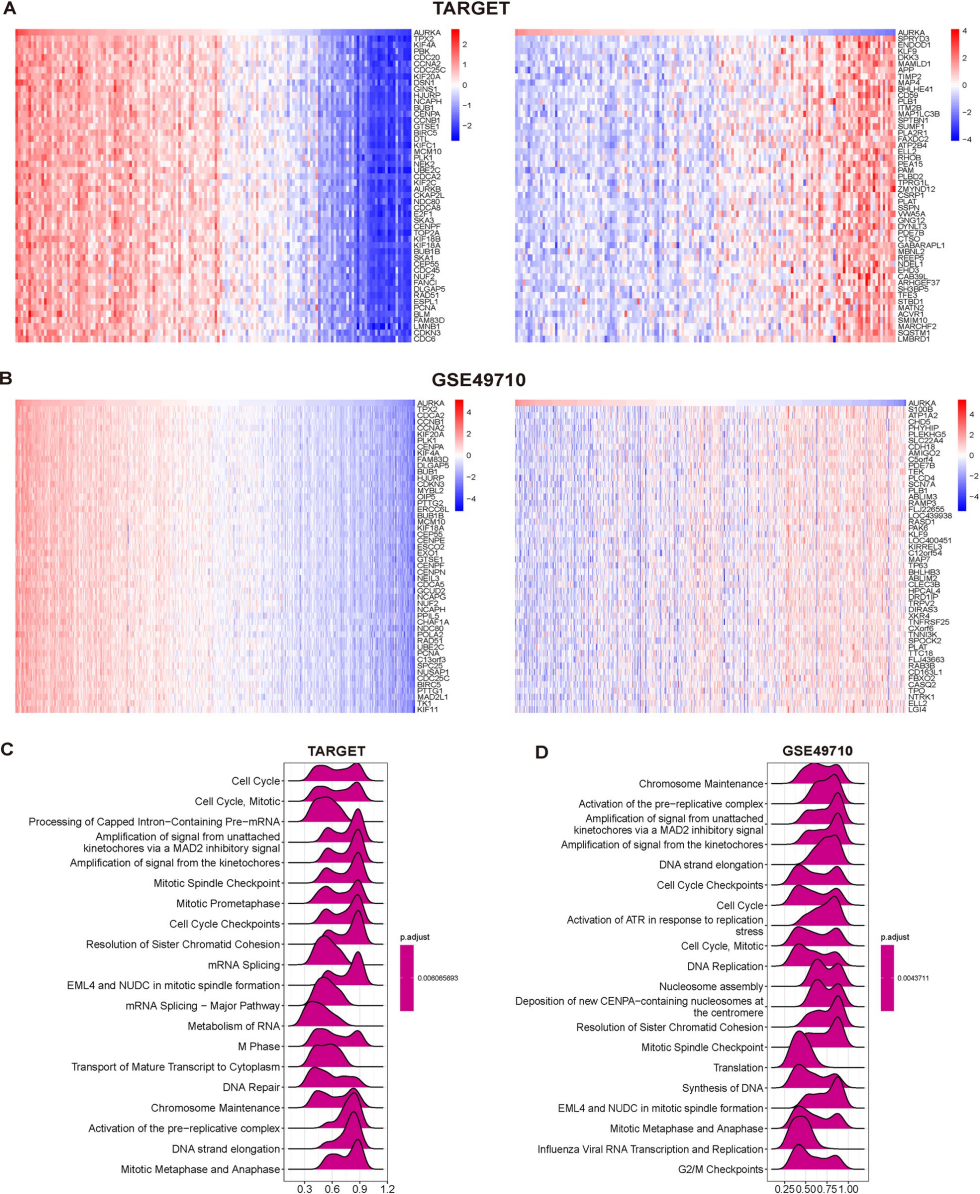
Correlation and functional enrichment analysis. (A) The heatmaps depicted the top 50 genes positively correlated to AURKA. (B) The heatmaps depicted the top 50 genes negatively correlated to AURKA. (C–D) Merged plots of GSEA indicating the signaling pathways associated with AURKA expression according to Reactome analyses in neuroblastoma.

To further explore the functions and pathways affected by AURKA, a correlation analysis between AURKA and all other mRNAs was performed using TARGET and GSE49710 datasets. The top 300 genes associated with AURKA were selected for enrichment analysis, and the top 50 genes that were either positively or negatively associated with AURKA are shown in the heatmap. In the TARGET database, TPX2, KIF4A, PBK, CDC20, and CCNA2 were the top five genes positively associated with AURKA; meanwhile, SPRYD3, ENDOD1, KLF9, DKK3, and MAMLD1 were the top five genes negatively associated with AURKA (Fig 2A). An analysis based on the GSE49710 dataset revealed that TPX2, CDCA2, CCNB1, CCNA2, and KIF20A were the top five genes positively associated with AURKA, whereas the top five genes negatively associated with AURKA were S100B, ATP1A2, CHD5, PHYHIP, and PLEKHG5 (Fig 2B).

The potential functional pathways were further explored based on the top 300 genes using the R “clusterProfiler” package. Based on the TARGET database, Reactome enrichment analysis based on the GSEA algorithm showed that AURKA was mainly involved in signaling pathways such as Cell Cycle, Mitotic, Processing of Capped Intron-Containing Pre-mRNA, Amplification of signal from the kinetochores, Mitotic Spindle Checkpoint, and Cell Cycle Checkpoints (Fig 2C). In the GSE49710 dataset, enriched pathways included Chromosome Maintenance, Activation of the pre-replicative complex, DNA strand elongation, Cell Cycle Checkpoints, among others (Fig 2D). These results suggest that AURKA is closely related to cell cycle-related pathways.

**Fig 3:**
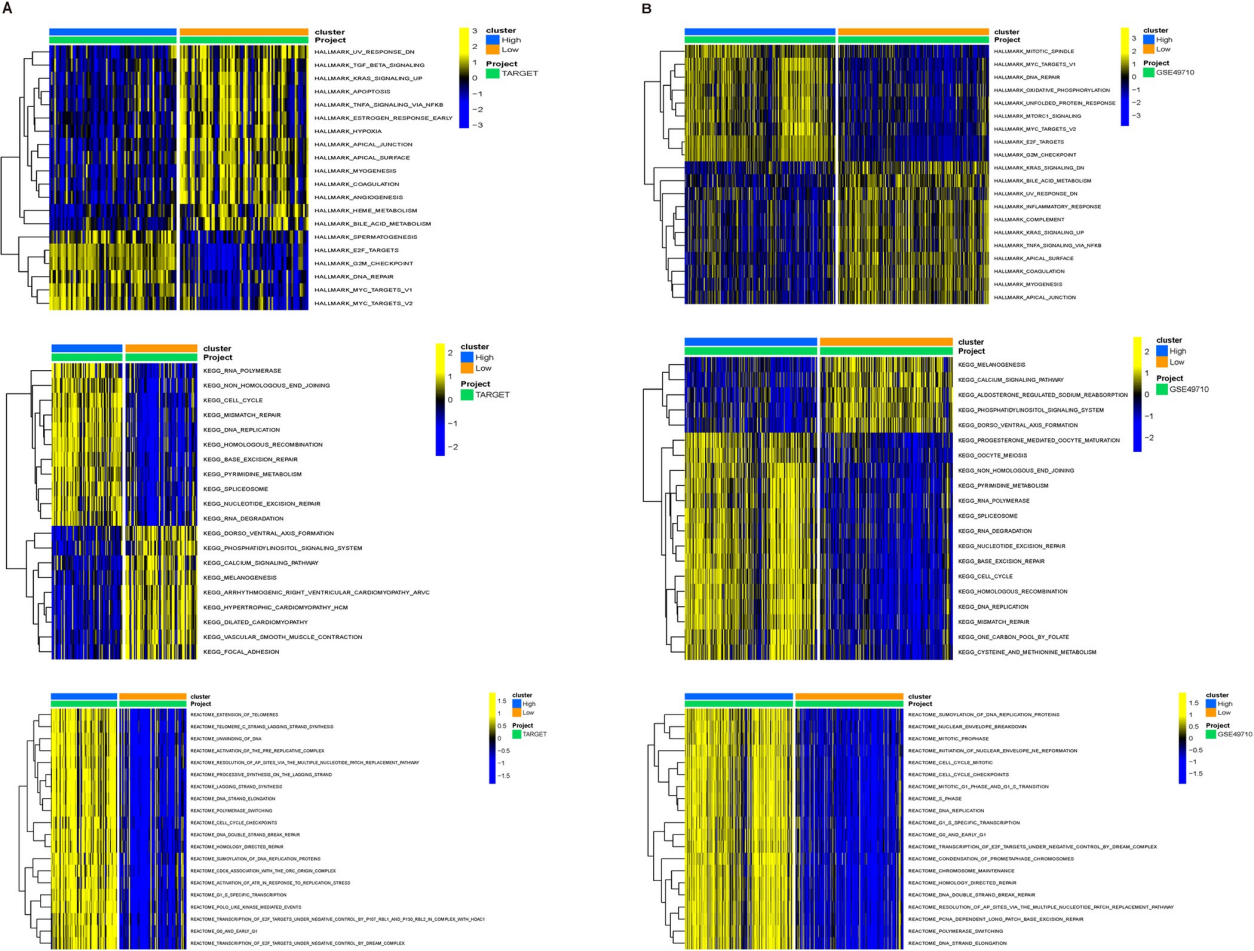
Results of GSVA. (A, B) Comparison of differences in pathways between AURKA-high- and low- expression group in neuroblastoma.

Subsequently, using the GSVA algorithm, a series of pathways and biological processes were identified enriched in AURKA to varying degrees. Based on the TARGET database, HALLMARK results indicated the significant enrichment of DNA repair and MYC-related pathways in the AURKA-high-expression group. KEGG results showed that high AURKA expression was significantly associated with the cell cycle, mismatch repair, and DNA replication-related pathways; Reactome results showed that the AURKA-high-expression group mainly exhibited gene enrichment in telomerase extension and cell cycle signaling pathways (Fig 3A). A further analysis of the GSE49710 dataset yielded similar findings, showing that MYC-related, DNA mismatch repair, cell cycle-related pathways, and the MTORC1 signaling pathway were significantly enriched in the AURKA-high-expression group (Fig 3B).

#### 1.3 AURKA reshapes the tumor immunosuppressive microenvironment

**Fig 4:**
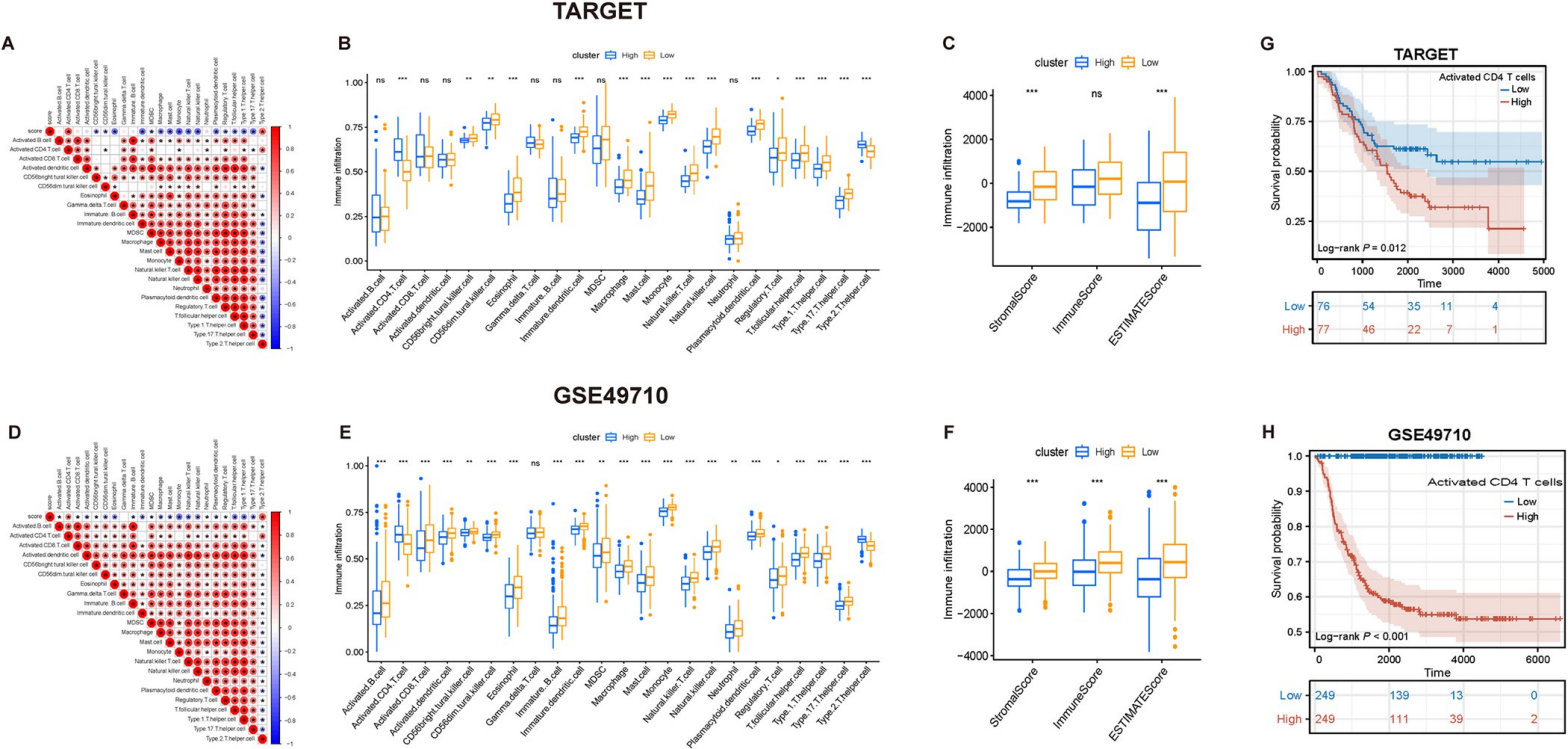
Correlation between AURKA expression and immune infiltration. (A, D) Single-sample Gene Set Enrichment Analysis (ssGSEA). (B, E) Abundance of each infltrating immune cell between AURKA-high- and low- expression group is shown in the boxplot. The median ± interquartile range of values is shown in the graph. ns P>0.05, *P < 0.05, **P < 0.01, ***P < 0.001. (C, F) The correlation of AURKA with Immune Score, Stromal Score and ESTIMATE score. (G, H) High levels of activated CD4 T cells infiltration predicted a poorer prognosis for patients with neuroblastoma.

Based on the TARGET database, the correlation between AURKA scores and immune cells was assessed, which showed that 15 of the 23 immune cells were negatively associated with AURKA scores (Fig 4A). Immune cell infiltration in the AURKA-high- and low-expression NB groups was further compared using the ssGSEA algorithm. The infiltration levels of 14 immune cells were significantly downregulated in the AURKA-high-expression group compared to those in the AURKA-low-expression group, with only activated CD4 T cells and type2 T helper cells showing increased infiltration in the AURKA-high-expression group (Fig 4B). The ESTIMATE algorithm showed that the stromal and ESTIMATE scores were significantly lower in the AURKA-high-expression group than in the AURKA-low-expression group (Fig 4C).

Based on the GSE49710 dataset, 20 immune cell types were negatively associated with the AURKA score (Fig 4D). With only activated CD4 T cells and type2 T helper cells showing significantly increased infiltration in the AURKA-high-expression group, in contrast, the infiltration levels of the remaining 20 immune cell types were significantly downregulated in this group (Fig 4E). Further, the stromal, immune, and ESTIMATE scores were all significantly lower in the AURKA-high-expression group than in the AURKA-low-expression group (Fig 4F). These results indicated that high AURKA expression was negatively associated with the immune activation status of NB. Moreover, Kaplan-Meier survival analysis showed that high levels of activated CD4 T cells infiltration predicted a poorer prognosis for patients with NB in TARGET (Fig 4G) and GSE49710 dataset (Fig 4H).

**Fig 5:**
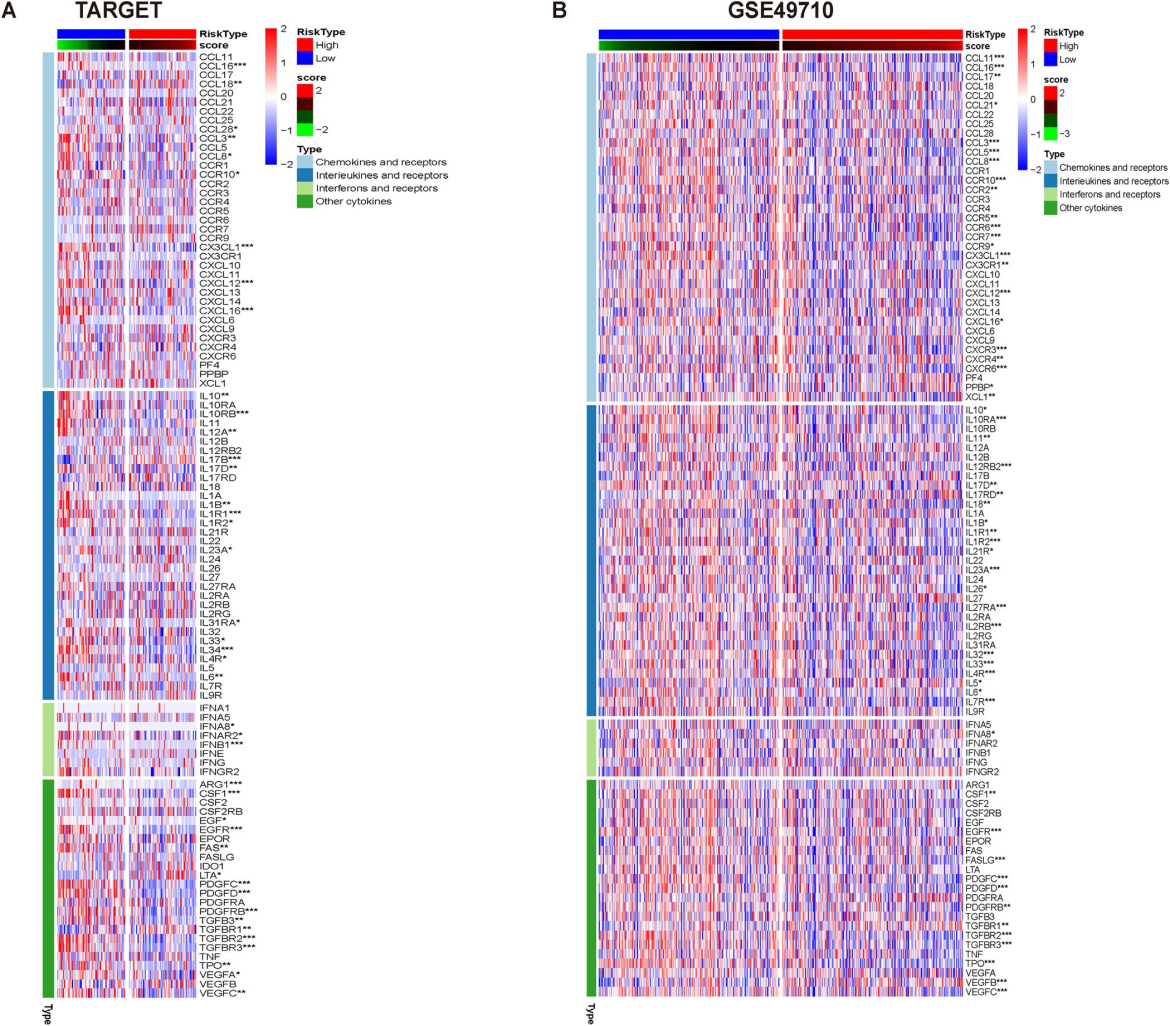
The association between AURKA and cytokines in neuroblastoma. (A) Based on the TARGET database. (B) Based on the GSE49710 dataset.

Based on the TARGET and GSE49710 datasets, the association between AURKA and chemokines and their receptors, interleukins and their receptors, interferons and their receptors, and other cytokines in NB was further analyzed using the TISIDB database. The results showed that in NB, AURKA was negatively associated with most cytokines in the afore-mentioned four categories (Fig 5A and 5B).

#### 1.4 Prediction of drug susceptibility for non-immunotherapeutic treatments

**Fig 6:**
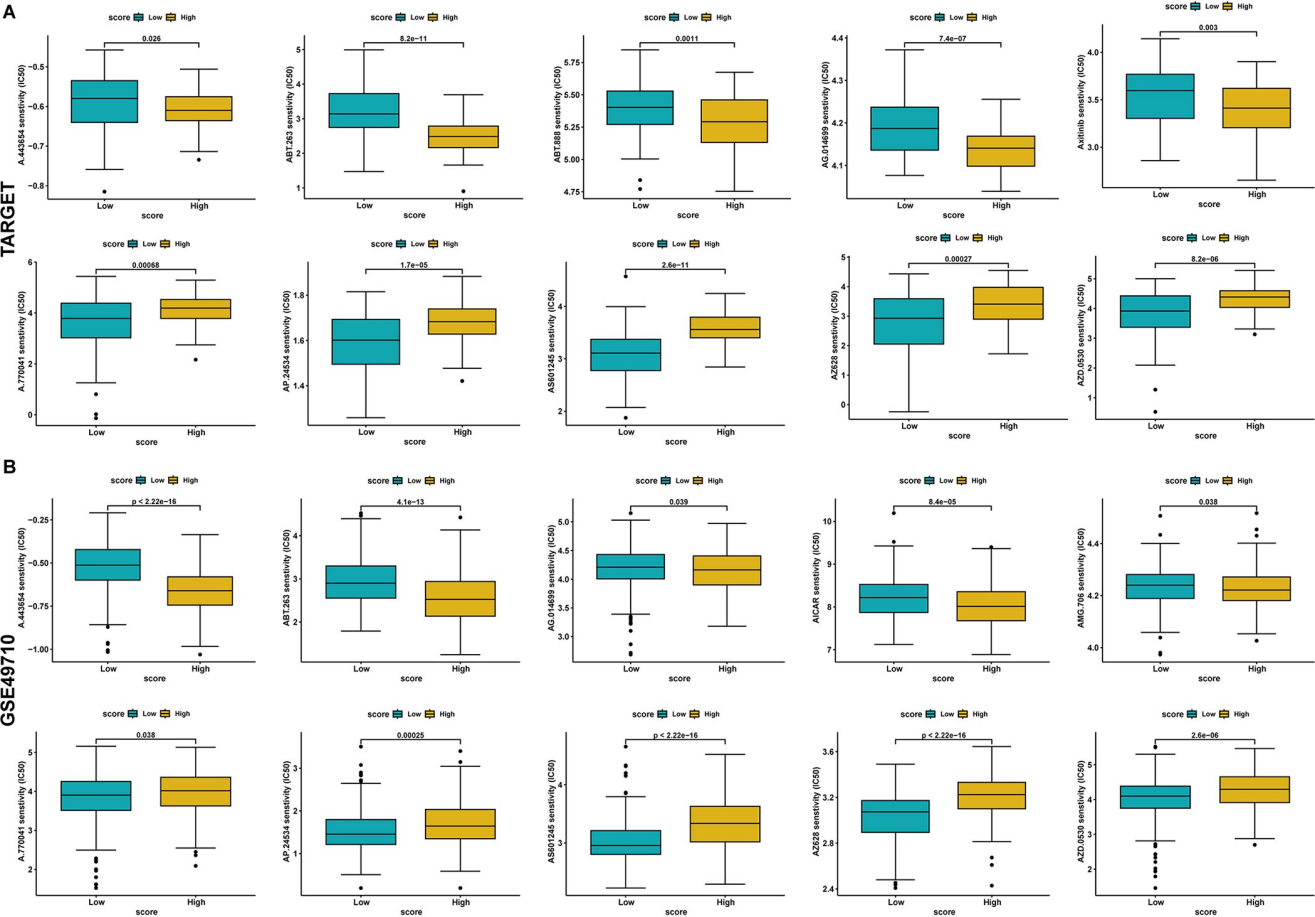
The correlation between the expression of AURKA and drug sensitivity. (A) Based on the TARGET database. (B) Based on the GSE49710 dataset.

The IC_50_ values for each sample, based on multiple anticancer drugs, were predicted using the R language pRRophetic package to compare the differences between the AURKA-high- and low-expression groups, with a higher IC_50_ indicating lower sensitivity to treatment. Analysis of the data using the TARGET database revealed sensitivity to the drugs A.443654, ABT.263, ABT.888, AG.014699 and Axitinib in the AURKA-high-expression group (Fig 6A). Moreover, according to the GSE49710 dataset, sensitivity to A.443654, ABT.263, AG.014699, AICAR and AMG.706 was apparent in the AURKA-high-expression group (Fig 6B).

### 2 IHC results

**Fig 7:**
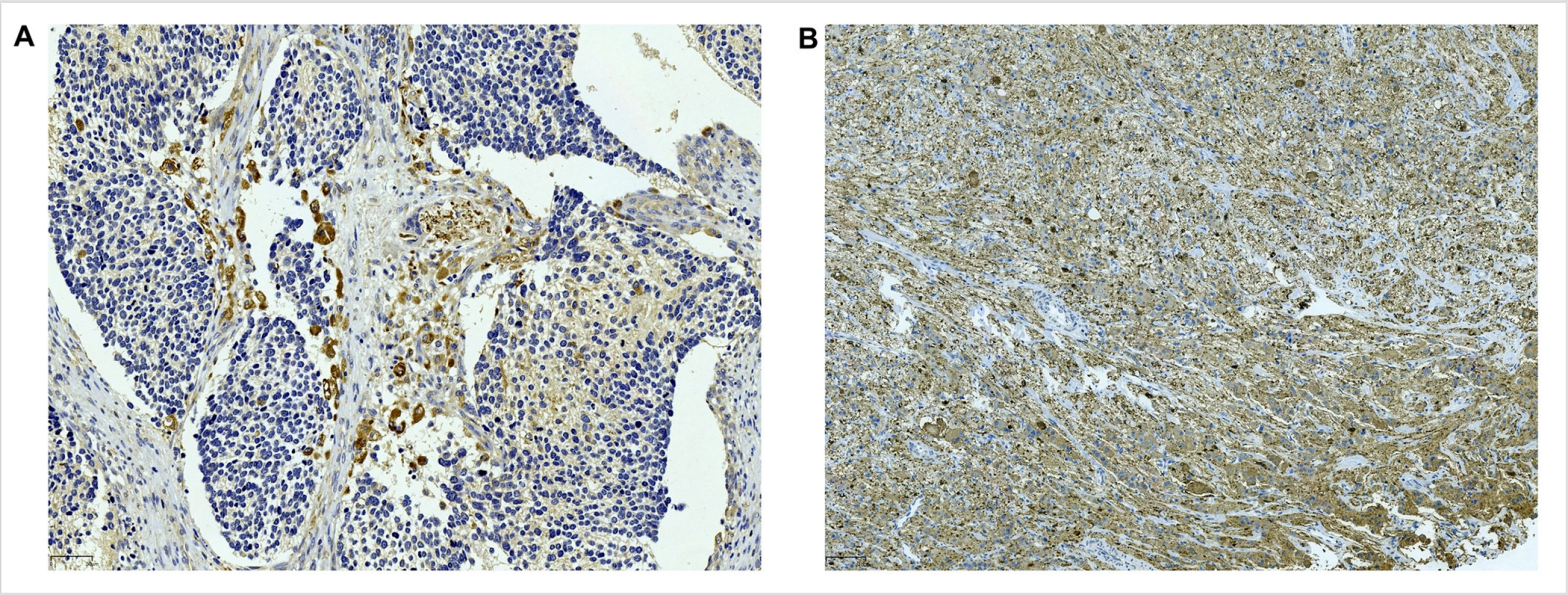
Immunohistochemical staining of AURKA in neuroblastoma. (A) High expression of AURKA (200×). (B) Low expression of AURKA (200×).

In total, 77 patients were included, consisting of 48 males and 29 females; 31 patients were diagnosed at ≤18 months of age, and 46 patients were diagnosed at >18 months. The origin of tumors was adrenal in 45 cases and extra-adrenal in 32 cases. Pathologically, 59 cases were classified as poorly differentiated, and 18 were undifferentiated; 29 cases had tumor MYCN amplification, whereas 48 cases did not. INSS (International Neuroblastoma Staging System) staging showed 50 cases of Stage I+II+IVs and 27 of Stage III+IV. COG risk grouping indicated that 32 cases had a low-intermediate risk and 45 cases had a high risk; 15 cases had high AURKA expression (Fig 7A), and 62 had low expression (Fig 7B).

Clinical data comparisons between the AURKA-high- and low-expression groups are represented in Table 1. The *χ*^*2*^ test showed no significant differences in proportions based on gender, age at diagnosis, primary tumor site, pathological type, and COG risk group between the AURKA high- and low-expression groups. However, the proportions of patients with MYCN amplification and stages III+IV disease were significantly higher in the AURKA high-expression group compared to those in the low-expression group, with all differences being statistically significant(*P*<0.01).

**Table 1:** Clinicopathologic characteristics of the patients with neuroblastoma and AURKA protein expression.

Clinical features	*n*	AURKA		*χ* ^*2*^	*P*
		Low expression(*n* = 62)	High expression(*n* = 15)		
**Gender**				0.643	0.423
Male	48(62.3)	40(64.5)	8(53.3)		
Female	29(37.7)	22(35.5)	7(46.7)		
**Age at diagnosis**				0.885	0.347
≤18 months	31(40.3)	29(46.8)	5(33.3)		
>18 months	46(59.7)	33(53.2)	10(66.7)		
**Primary site**				0.130	0.718
Extra-adrenal	32(41.6)	28(45.2)	6(40.0)		
Adrenal	45(58.4)	34(54.8)	9(60.0)		
**Pathological type**				1.031	0.310
undifferentiated	18(23.4)	13(20.97)	5(33.3)		
poor-differentiated	59(76.6)	49(79.03)	10(66.7)		
**MYCN status**				6.675	0.010
Non-amplified	48(61.3)	43(60.7)	5(33.3)		
Amplified	29(38.7)	19(39.3)	10(66.7)		
**INSS stage**				11.982	<0.001
I+II+IVs	50(26.7)	46(25.0)	4(31.6)		
III+IV	27(73.3)	16(75.0)	11(68.4)		
**COG risk group**				0.200	0.655
Low+Intermediate	32(41.6)	25(40.3)	7(46.7)		
High	45(58.4)	37(59.7)	8(53.3)		

With the expression of AURKA in patients as the dependent variable and the variables with a *P-*value <0.05 in the univariate analysis as the independent variables (including the MYCN status and INSS stage), a multifactorial logistic regression model was established using a stepwise method. The results showed that MYCN status and INSS stage were not independent factors affecting AURKA expression (Table 2).

**Table 2:** Logistic regression analysis of the correlation between AURKA expression and clinical pathological characteristics.

Characteristics	*B*	*SE*	*Waldχ* ^*2*^	*P*	*OR (95% CI)*
INSS stage	0.983	0.859	1.310	0.252	2.672(0.496, 14.386)
MYCN status	0.157	0.623	0.064	0.801	1.170(0.345, 3.963)
Constant (quantity)	-2.259	0.744	9,216	0.002	-

The log-rank test showed that pathological type, MYCN status, INSS stage, COG risk group, and AURKA expression was correlated with PFS of patients(*P*<0.01) (Table 3). A COX proportional hazards model was established with disease progression or patient death as the dependent variable (yes = 1, no = 0) and pathological type, MYCN status, INSS stage, COG risk group, and AURKA expression as the independent variables. The results showed that the above factors were not independently prognostic of PFS (Table 4).

**Table 3:** Clinicopathologic characteristics of the patients with neuroblastoma and progression-free survival (PFS).

Patient characteristics	n	PFS Mean (95% CI)	log-rank*χ*^*2*^	*P*
**Gender**			1.963	0.161
Male	48(62.3)	79.702(67.335, 92.069)		
Female	29(37.7)	53.525(40.250, 64.800)		
**Age at diagnosis**			0.454	0.500
≤18 months	31(40.3)	62.049(51.182, 72.916)		
>18 months	46(59.7)	70.620(56.994, 84.246)		
**Primary site**			0.460	0.498
Extra-adrenal	32(41.6)	61.323(50.391, 72.255)		
Adrenal	45(58.4)	71.145(57.434, 84.855)		
**Pathological type**			13.065	<0.001
undifferentiated	18(23.4)	31.078(20.571, 41.585)		
poor-differentiated	59(76.6)	82.238(71.480, 92.995)		
**MYCN status**			25.204	<0.001
Non-amplified	48(61.3)	94.228(84.799,103.656)		
Amplified	29(38.7)	36.136(25.822, 46.449)		
**INSS stage**			10.750	0.001
I+II+IVs	50(26.7)	80.429(73.597, 87.260)		
III+IV	27(73.3)	62.185(49.669, 74.701)		
**COG risk group**			21.536	<0.001
Low + intermediate	32(41.6)	104.70(98.341, 111.059)		
High	45(58.4)	44.013(34.676, 53.350)		
**AURKA**			8.499	0.004
Low expression	62(80.5)	81.072(70.209, 91.935)		
High expression	15(19.5)	37.689(22.839, 52.538)		

**Table 4:** Univariate Cox analysis of potential risk factors for poor progression-free survival of neuroblastoma patients.

Covariate	*B*	*SE*	*Waldχ* ^*2*^	*P*	*HR (95% CI)*
Pathological type	0.507	0.424	1.435	0.231	1.661(0.724, 3.810)
MYCN status	0.837	0.472	3.149	0.076	2.310(0.916, 5.826)
INSS stage	0.685	1.169	0.343	0.558	1.984(0.201, 19.621)
COG risk group	2.054	1.225	2.811	0.094	7.796(0.707, 85.997)
AURKA	0.558	0.430	1.687	0.194	1.747(0.753, 4.056)

### 3 Cell experiments in vitro

#### 3.1 Targeted knockdown of AURKA induces growth inhibition, G2/M phase blockade, and apoptosis in NB cell line

**Fig 8:**
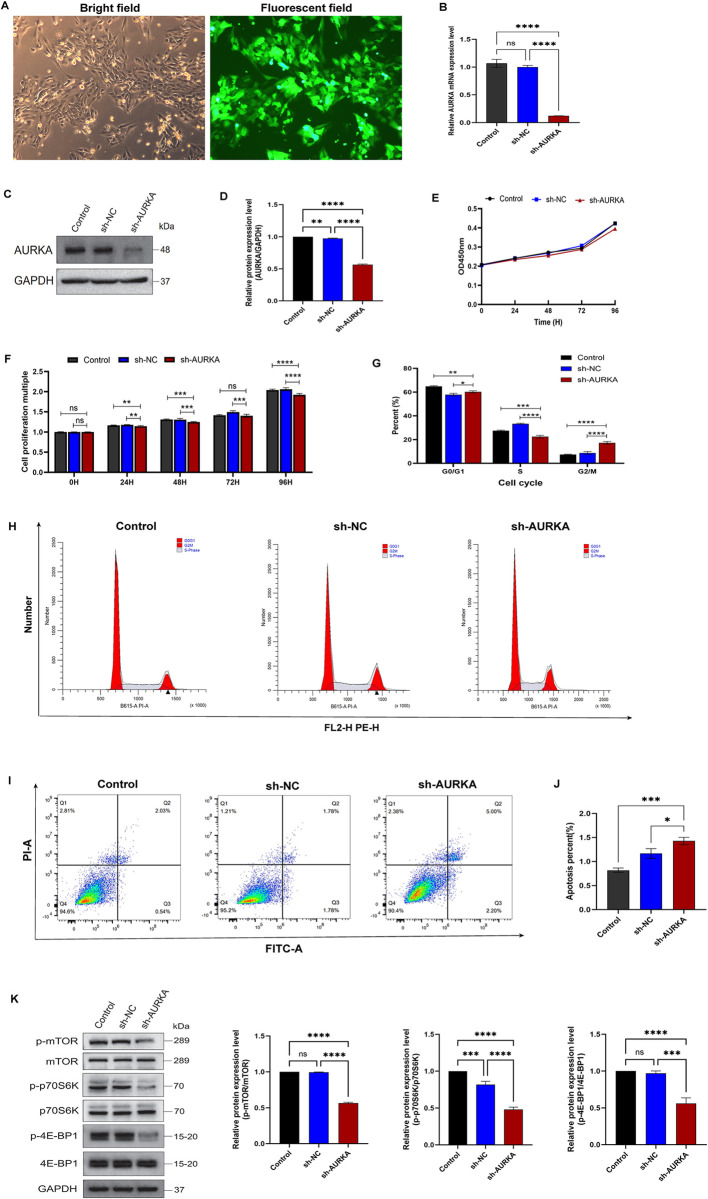
Targeted knockdown of AURKA induces growth inhibition, G2/M phase blockade, and apoptosis in NB cell line. (A) At MOI of 10, lentivirus transduction efficiency was estimated three days after infection (original magnification, ×200). (B) RT-qPCR of the expression of AURKA mRNA. (C, D) Western blotting of the expression of AURKA protein. (E, F) CCK8 cell proliferation experiment after interfering AURKA expression using shRNA in SK-N-AS cells. (G, H) Cell cycle analysis of three cell groups was detected by PI staining and FACS analysis in SK-N-AS cells. (I) Flow cytometry profile represents Annexin-V-FITC staining in the x-axis and PI in the y-axis. (J) AURKA-specific shRNA induced cell apoptosis in SK-N-AS cells. (K) Representative images of protein expression examined by Western blot. GAPDH was used as a normalization control. Bars graphs show quantifications for the indicated proteins. Data are expressed as the mean ± standard deviation (SD) obtained from 3 dependent experiments. (*P < 0.05, **P < 0.01, ***P < 0.001, ****P < 0.0001). Control, uninfected cells; sh-NC, cells infected with control lentivirus; sh-AURKA, cells infected with AURKA-shRNA lentivirus. NB, Neuroblastoma; PI, propidium iodide; FACS, fluorescence-activated cell sorting; FITC, fluorescein isothiocyanate.

Recombinant lentiviruses delivered shRNA to SK-N-AS cells to silence AURKA expression were used to study the role of AURKA in NB. The infection efficiency of AURKA-shRNA lentiviruses was >80% (Fig 8A). The expression of AURKA mRNA (Fig 8B) and protein (Fig 8C and 8D, S1 Raw images) was effectively inhibited in the sh-AURKA group, compared with that in the sh-NC and control (uninfected cells) groups. CCK-8 assay was further performed to examine the effect of AURKA on the cell proliferation rate, with results indicating a reduced proliferative capacity in the sh-AURKA group at 48, 72, and 96 h, compared to that in the control and sh-NC groups (Fig 8E and 8F).

Flow cytometric analysis showed that the proportion of G2/M-phase cells was significantly higher and the proportion of S-phase cells was significantly lower in the sh-AURKA group compared with those in the control and sh-NC groups, and the differences were all statistically significant (Fig 8G and 8H). Cell apoptosis in the control, sh-NC, and sh-AURKA groups was detected using Annexin-FITC and PI double staining, and the results showed that cell apoptosis rates in the control, sh-NC, and sh-AURKA groups were 0.82±0.04%, 1.17±0.10%, and 1.43±0.08%, respectively. Moreover, the rate of SK-N-AS cell apoptosis was increased after AURKA knockdown (Fig 8I and 8J).

In the part of the biological information analysis using the GSVA algorithm, the results showed that the MTORC1 signaling pathway were significantly enriched in the AURKA-high-expression group. Therefore, western blotting was used to detect MTORC1 pathway downstream protein expression levels and observed that the phosphorylation levels of mTOR, p70S6K, and 4EBP1 proteins were significantly downregulated in the sh-AURKA group (Fig 8K, S1 Raw images). This indicated that silencing AURKA might affect NB cell biological functions through the inhibition of mTORC1/p70S6K/4E-BP1 pathway activity.

#### 3.2 Constructing a PPI network and miRNA-TFs -AURKA network diagrams

**Fig 9:**
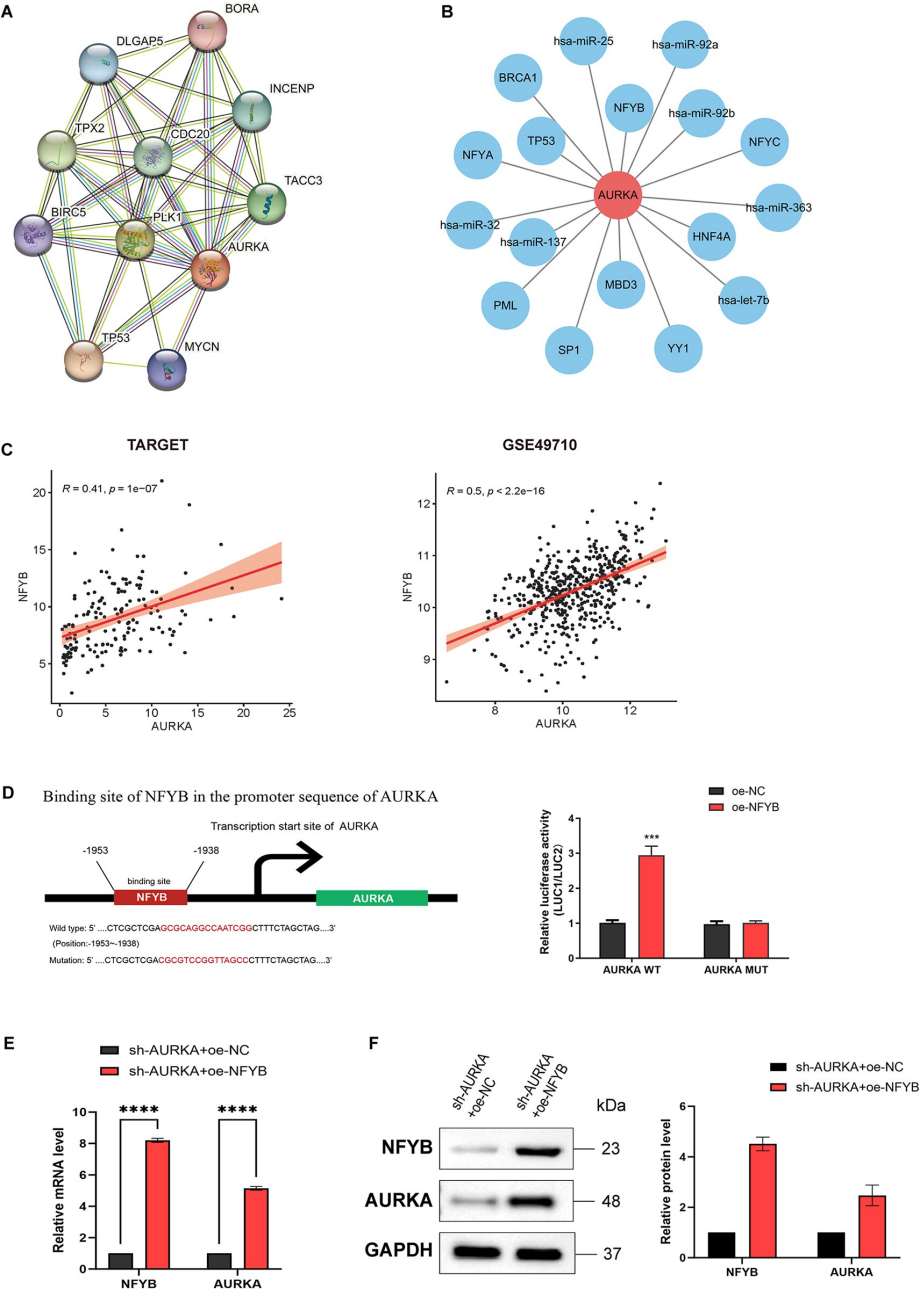
Constructing a PPI network and miRNA-transcription factors-AURKA network diagrams. (A) The PPI was predicted for the AURKA-encoded protein, with network nodes representing proteins, connecting lines representing predicted relationships, light blue representing auxiliary database evidence, purple representing experimental validation, yellow representing text mining evidence, green representing gene proximity, red representing gene fusions, blue representing gene co-occurrence, black lines representing gene co-expression, and grey lines representing protein homology. (B) miRNA-transcription factors-AURKA network diagrams. (C) Spearman’s correlation analysis of AURKA with NFYB expression in the TARGET and GSE49710 database; (D) The binding of NFYB to the AURKA promoter verified by luciferase reporter assay; The expression of NFYB and AURKA in SK-N-AS cells after co-transfection detected by RT-qPCR (E) and western blot (F).

The STRING database was used to construct a PPI network based on AURKA, and the results showed that AURKA interacts with MYCN, TP53, BIRC5(survivin), PLK1, TACC3, CDC20, TPX2, INCENP, DLGAP5, and BORA (Fig 9A). BORA is an activator of AURKA, and the BORA-AURKA complex is a major activator of PLK1 during mitosis. The combined action of the three genes promotes cell entry into mitosis, which could contribute to the strong proliferative potential of malignant tumor cells when AURKA is overexpressed [26]. MYCN, TP53, and BIRC5 are all key molecules involved in the development of NB [27]. As such, high PLK1 [28], TPX2 [29], and INCENP [30] expression levels are all associated with poor prognosis in patients with NB.

Then miRNAs and TFs upstream of AURKA was predicted in the RegNetwork database, identifying 7 miRNAs (has-miR-137, has-miR-92b, has-miR-92a, has-miR-25, has-miR-32, has-miR-363, has-let-7b) and 10 TFs (TP53, HNF4A, MBD3, YY1, SP1, PML, NFYA, NFYB, NFYC, BRCA1) as regulators upstream of AURKA (Fig 9B). Thus, I analyzed the correlation of AURKA with the expression of TFs in the TARGET and GSE49710 dataset by Spearman’s correlation. It was revealed that AURKA had the significant positive association with NFYB (Fig 9C).

Wild-type pGL4.10 luciferase reporter vector containing AURKA promoter and the corresponding mutant vector were constructed, and transfected NFYB overexpression (oe) plasmid into SK-N-AS cells, respectively. The results showed that the luciferase activity was significantly increased in the cells transfected with oe-NFYB plasmid and AURKA wild-type vector, while the luciferase activity was not significantly changed in the cells with mutant or oe-NC (Fig 9D).

Furthermore, I overexpressed NFYB in SK-N-AS cells that already had stable low expression of AURKA. The expression of AURKA and NFYB in the cells had a significant increase after co-transfection (Fig 9E and 9F, S1 Raw images). The above results suggest that AURKA was transcriptionally regulated by NFYB.

## Discussion

The prognosis of high-risk patients with NB is poor, as tumors continue to progress even after multidisciplinary combination therapy. Identifying genes and molecular markers associated with NB progression and understanding their potential mechanisms in NB is a hot topic in current research, as this will provide a stronger evidence base for personalized treatment. Research has shown that the expression level of AURKA is upregulated in a variety of human cancers, including gastric, colorectal, hepatocellular, ovarian, renal, bladder, and prostate cancers, and that the high expression of AURKA is associated with clinical aggressiveness, poor prognosis, and treatment resistance, suggesting that AURKA has become an important biomarker and therapeutic target for the diagnosis and prognostic prediction of different cancers [31]. The results of this study based on the TARGET and GSE49710 datasets showed that patients with NB exhibiting high expression of AURKA had a shorter OS. IHC was used to detect the expression of AURKA in NB tumor tissues from 77 patients. The results showed that AURKA was positively located in the cytoplasm and nucleus of tumor cells. Upon further analysis in conjunction with clinical data, it was found that the expression of AURKA protein was associated with MYCN status and INSS stage. Pathological type, MYCN status, INSS stage, COG risk group, and AURKA expression was correlated with PFS of NB patients. The literature indicates that age at diagnosis, pathological type, and MYCN status are independent prognostic factors for NB patients [32,33]. Furthermore, Ramani et al. [4] discovered that high expression of AURKA serves as an independent prognostic factor for OS and event-free survival in patients with NB. However, independent factors affecting the prognosis of NB patients were not found through COX regression analysis in this article, which may be due to the small number of NB cases in this study. Further research with a larger sample size is needed to clarify this issue.

AURKA knockdown significantly inhibited bladder, gastric, and nasopharyngeal cancer cell growth [34]. In NB, MYCN gene amplification predicted poor patient prognosis and treatment resistance. AURKA forms a complex with the N-Myc protein encoded by the MYCN gene and protects N-Myc from proteasomal degradation mediated by the Fbxw7 ubiquitin ligase [35]. In addition, structural stabilization of N-Myc proteins mediated by AURKA further upregulated VEGF expression in NB and promoted intra-tumor angiogenesis [36]. Since MYCN amplification was only detected in approximately 20% of NB cases, the biological function of AURKA has not been fully elucidated in non-MYCN-amplified NB. Therefore, SK-N-AS, a non-MYCN-amplified human NB cell line, was selected in this study to explore the role of AURKA in the regulation of tumor progression. Endogenous AURKA expression in SK-N-AS cells was specifically inhibited using an AURKA-shRNA lentivirus. Silencing AURKA in NB cell lines resulted in diminished proliferation and promoted tumor cell apoptosis. Further, AURKA overexpression promoted cell cycle progression in the presence of DNA damage or chromosome segregation abnormalities, leading to genomic and chromosomal instability, a hallmark of malignant tumors. For example, high levels of AURKA expression significantly enhance the dephosphorylation of CDK1, which promotes the G2/M phase transition of the cell cycle via the p53, PLK1 and CDC25 pathways [27]. In this study, silencing AURKA arrested the cell cycle in the G2/M phase in NB tumor cells, as determined via flow cytometry.

Next, the potential mechanisms by which AURKA affects NB progression was explored in this study through GSVA analysis. The activation of many pathways associated with tumor progression was significantly upregulated in the AURKA-high-expression group compared to that in the AURKA-low-expression group, and these included the cell cycle, mTORC1 signaling, and MYC_TARGETS_V2. Selective telomere lengthening, observed in approximately 20–25% of high-risk NB cases, was indicative of a poor prognosis [37]. Moreover, mTOR (mammalian target of rapamycin), a downstream protein kinase of the PI3K/AKT signaling pathway, plays an important role in physiological and pathological processes, such as cell proliferation, differentiation, autophagy, protein synthesis, and tumor growth. mTOR interacts with other proteins to form a complex, namely mTORC1. The two downstream signaling molecules of mTORC1 are p70S6K and 4E-BP1. In addition, the activation of 4E-BP1 and p70S6K via mTORC1 can promote protein synthesis [38]. Based on *in vitro* cytological experiments, the targeted knockdown of AURKA in the SK-N-AS cell line could affect the biological behavior of NB cells by inhibiting the mTOR/p70S6K/4E-BP1 signaling pathway.

Complex interactions exist between tumor cells and the TME in NB. The TME, consisting of a variety of immune cells, mesenchymal-derived cells, and the extracellular matrix, influences tumorigenesis and development through direct interactions with tumor cells. Immune cells infiltrating the TME are important factors affecting tumor growth, progression, therapeutic efficacy, and patient prognosis [39]. AURKA showed a negative correlation with the infiltration of B cells, CD8+ T cells, and T cell regulatory cells, but a positive correlation with CD4+ T cell Th2 in nasopharyngeal carcinoma [40]. In this study, high AURKA expression in NB was also negatively correlated with the level of infiltration of 20 immune cells and positively correlated only with the level of infiltration of activated CD4+ T cells and T helper 2 cells. Further analysis revealed that high levels of CD4+ T cell infiltration were significantly correlated with a poor prognosis of patients with NB. This suggests that AURKA might be involved in tumor immune evasion, leading to a poor prognosis for patients with NB, and that AURKA inhibition could be helpful to improve the immunotherapeutic efficacy for NB.

Drug sensitivity analysis was performed on AURKA. The result showed that A.443654, ABT.263, AG.014699, Axitinib et al. were the sensitive drugs in the AURKA-high-expression group. Among them, A.443654, a specific Akt inhibitor, interferes with mitotic progression and bipolar spindle formation. Akt inhibition attenuates mitotic arrest and defects in bipolar spindle formation when AURKA is overexpressed in cells treated with A.443654 [41]. Bcl-2 family members (Bcl-2, Bcl-xL and Bcl-w) are antagonized by ABT-263, a new BH3 mimetic [42]. Selective small-molecule inhibitors of BCL-XL may enhance the efficacy of MLN8237(an Aurora kinase inhibitor) and other targeted chemotherapeutic agents in medulloblastoma and pediatric glioblastoma cells [43]. Therefore, I speculate that these AURKA-related drugs can be applied to the treatment of NB.

This study had certain limitations. First, cell lines were solely used for experimental validation, without employing tumor-bearing mouse models. Therefore, animal studies are needed to further clarify the role of AURKA in NB. Second, although the correlation between AURKA and immune cell infiltration in NB was explored in this study, experimental evidence to validate the role of AURKA in regulation of the TME of NB was lacking.

In conclusion, by using bioinformatics and basic experiments, the expression, function, and molecular mechanism of AURKA in NB were systematically elucidated in this study, confirming that high expression is significantly associated with a poor prognosis for patients with NB. Moreover, silencing AURKA in an *in vitro* NB cell line was observed to affect the biological function of these cells through inhibition of the mTORC1 pathway. In addition, AURKA was observed to be negatively associated with host immune cell infiltration. This work further confirmed that AURKA is a diagnostic marker and therapeutic target for NB.

## Supporting information

S1 File The processed raw data of TARGET database. (CSV)

S2 File The group division of TARGET database. (XLSX)

S3 File The processed raw data of GEO database. (CSV)

S4 File The group division of GEO database. (XLSX)

S1 Fig The workflow chart of the study. (TIF)

S1 Raw images Original images of western blot. (PDF)
