# Supplementary figures and images for: Validation of the biological function and prognostic significance of AURKA in neuroblastoma
Source: PLoS One. 2024 Nov 25;19(11):e0313939. doi: 10.1371/journal.pone.0313939 (PMC11588284; doi:10.1371/journal.pone.0313939)

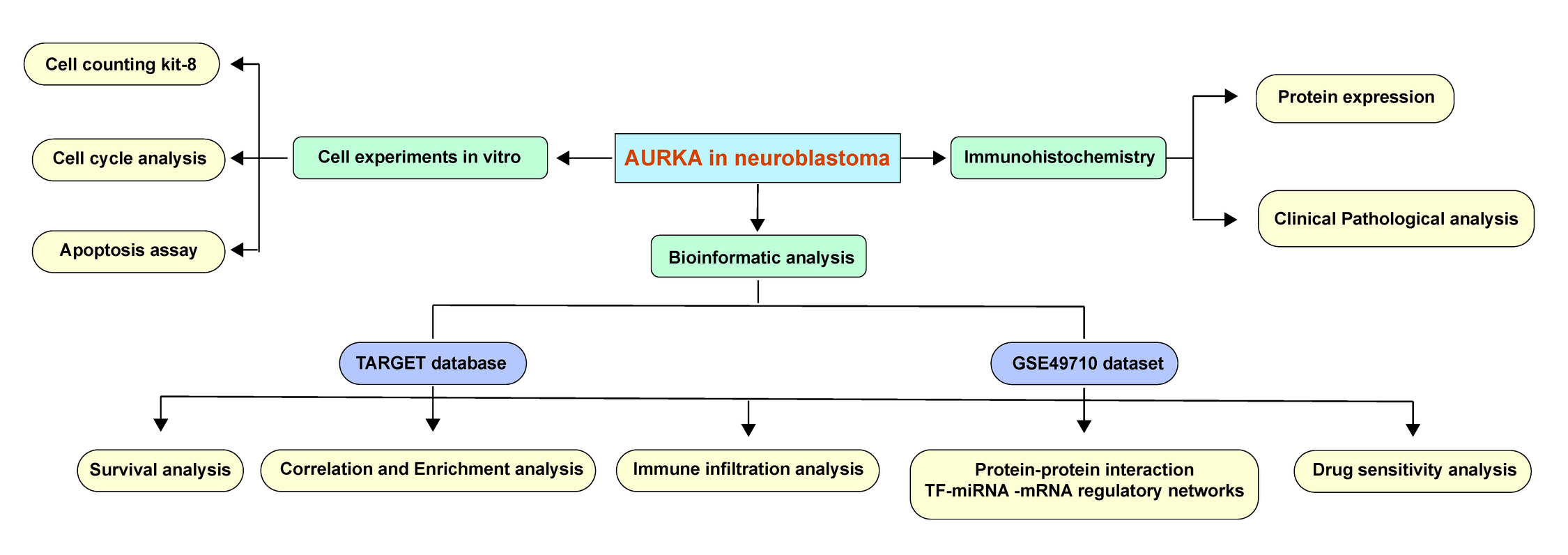

Supplement: S1 Fig — (TIF) [file pone.0313939.s005.tif]

Original images of western blot

Figure 8C

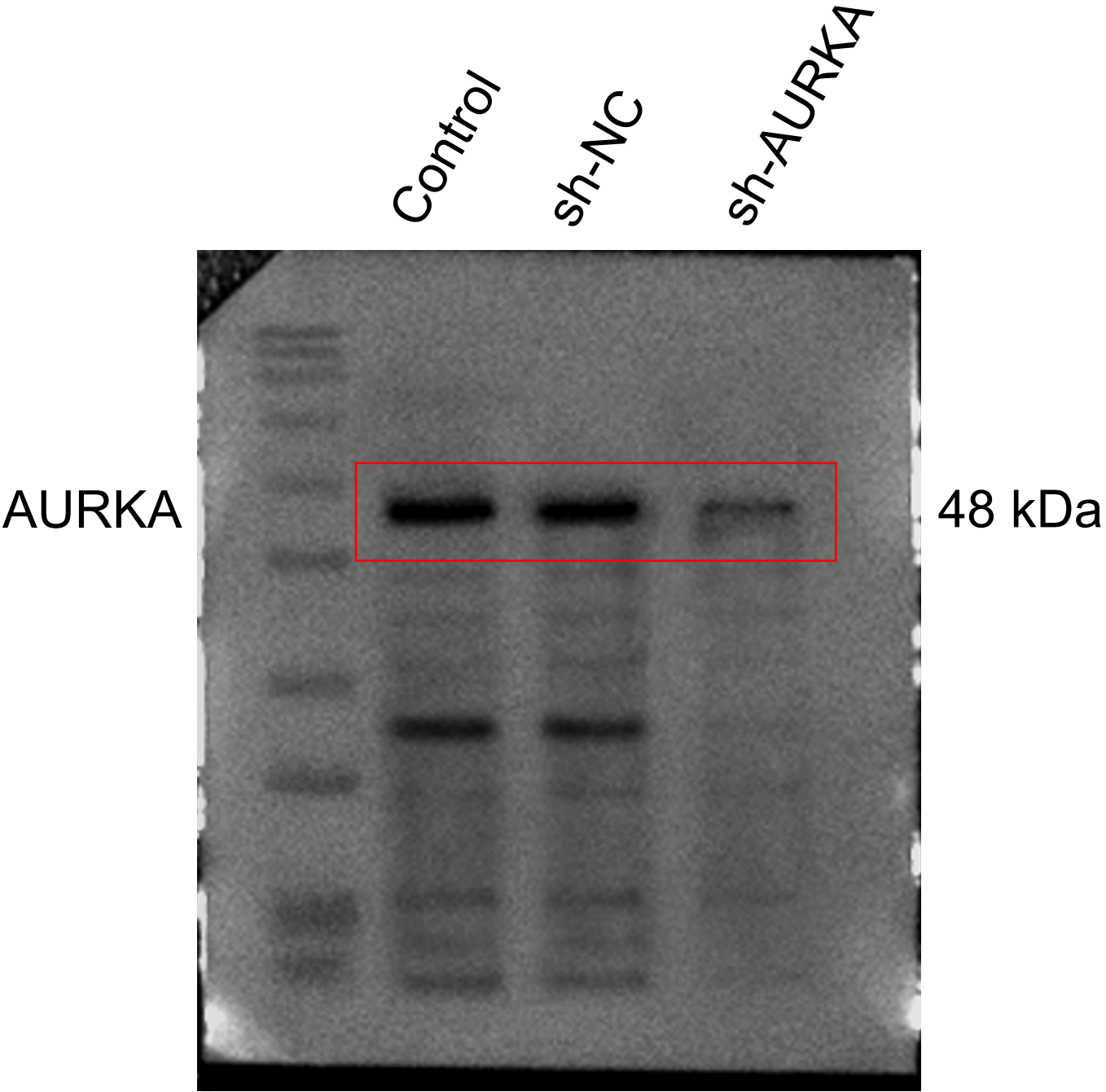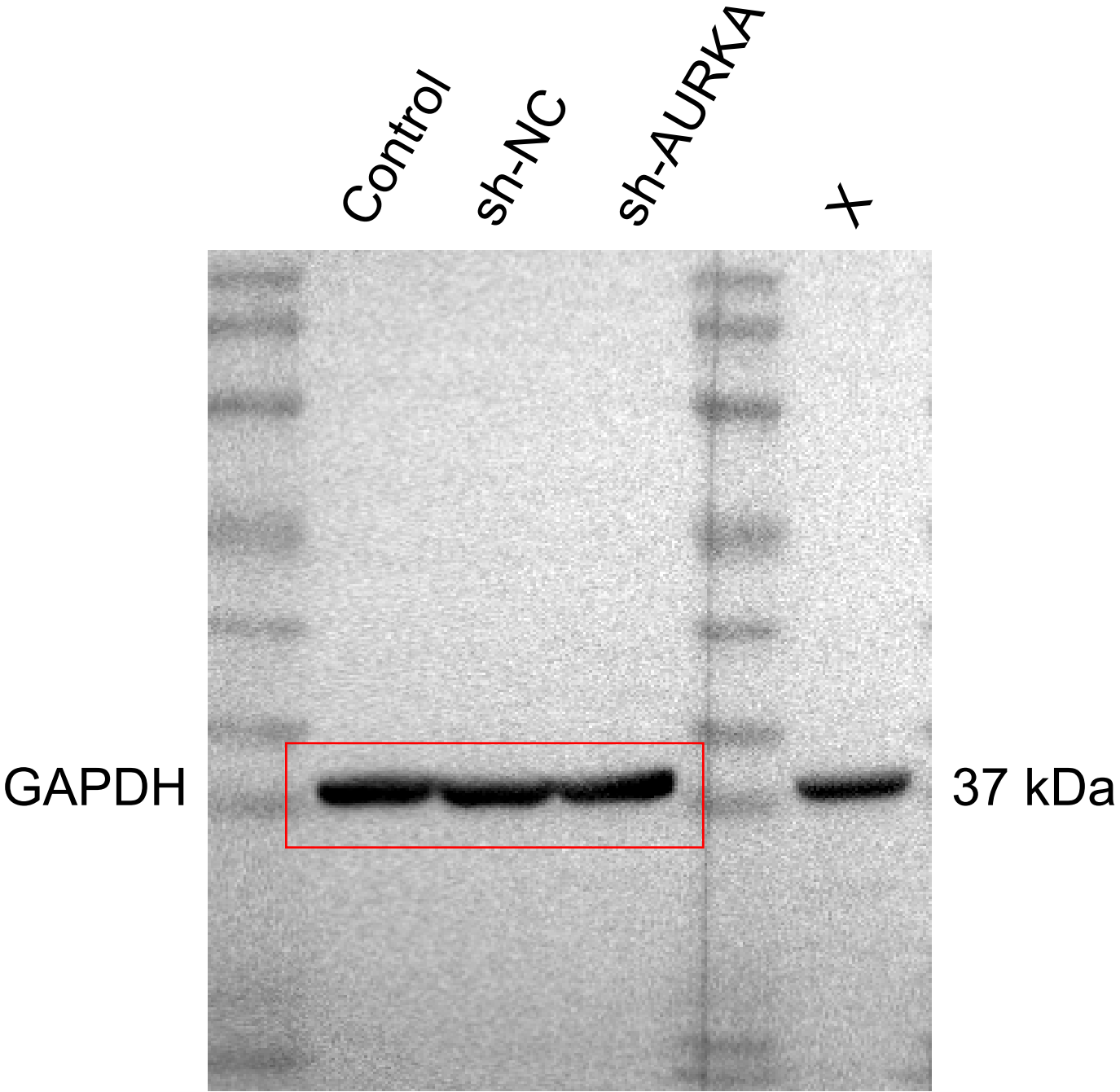

Figure 8K

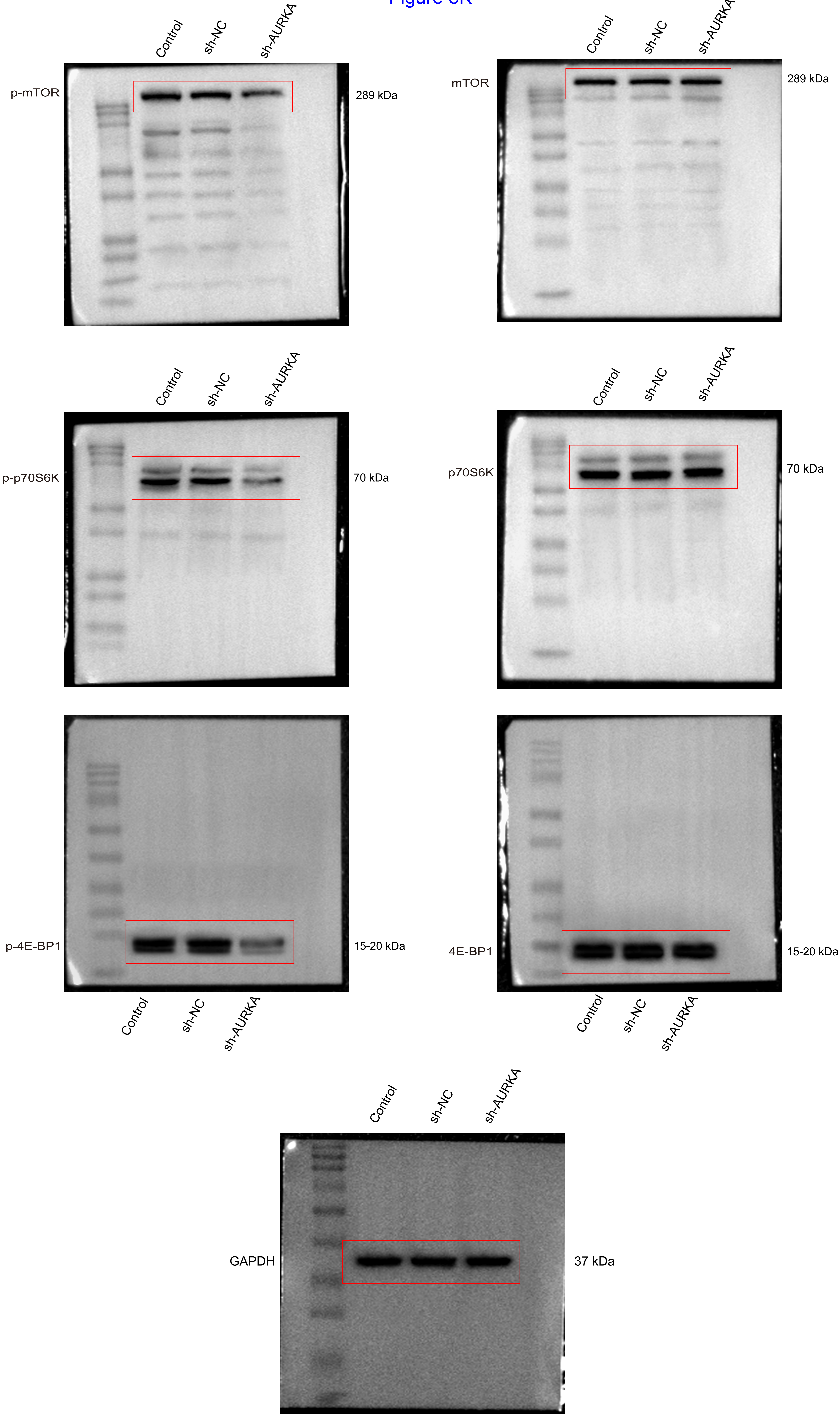

Figure 9F

AURKA

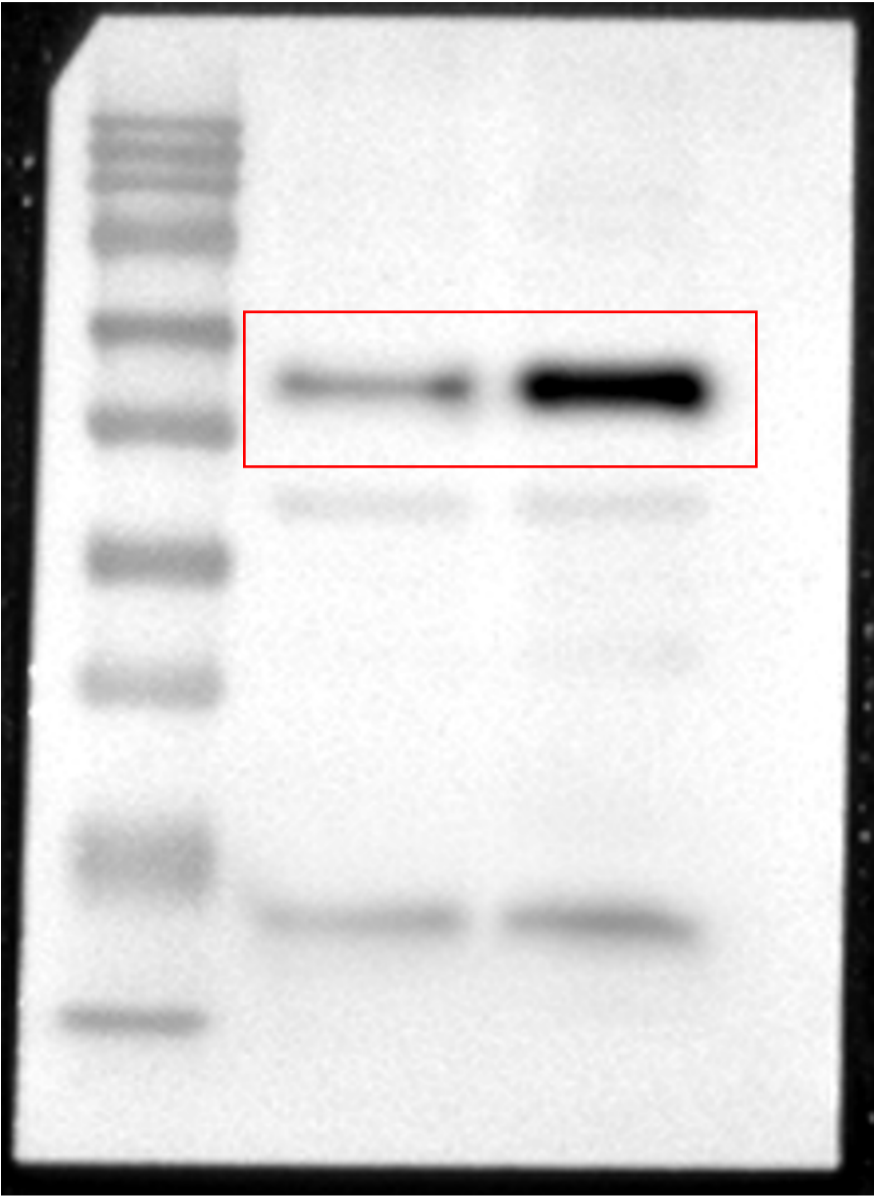

48 kDa

NFYB

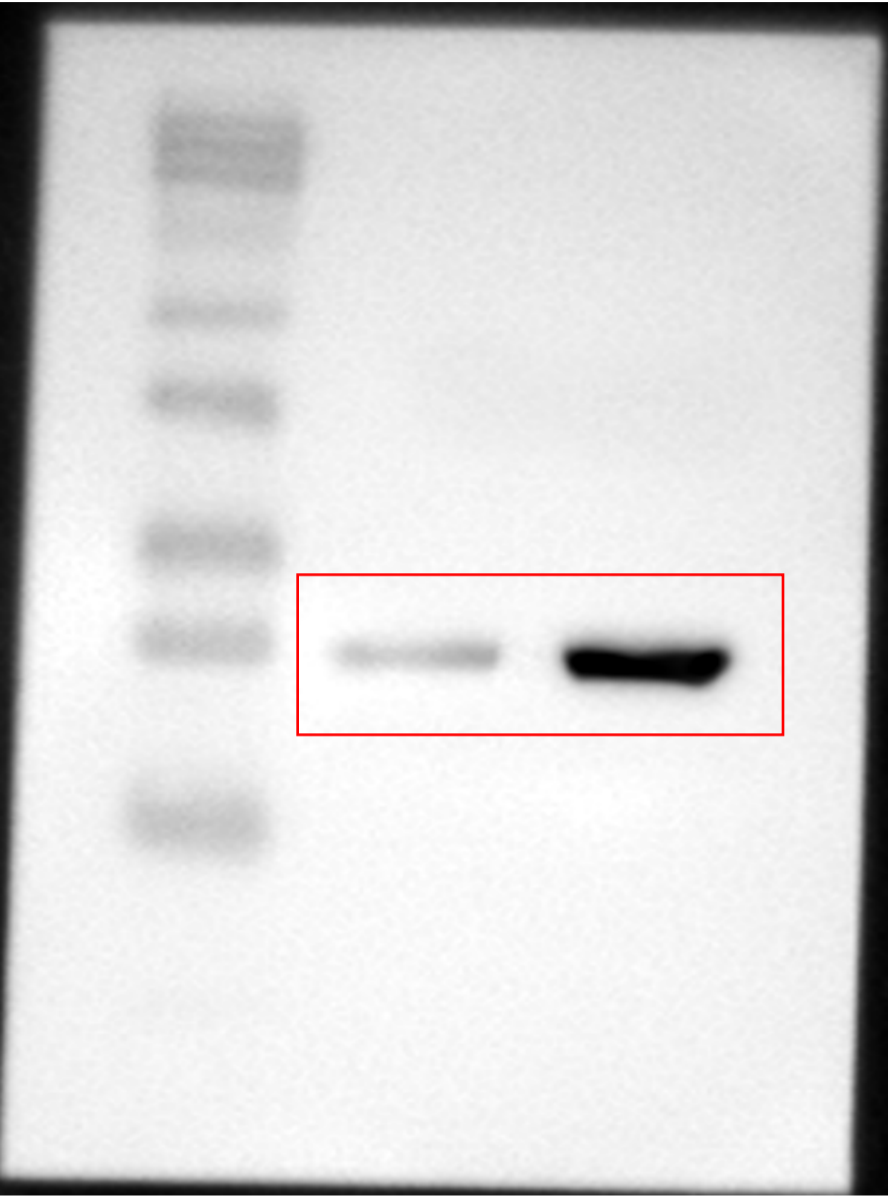

23 kDa

GAPDH

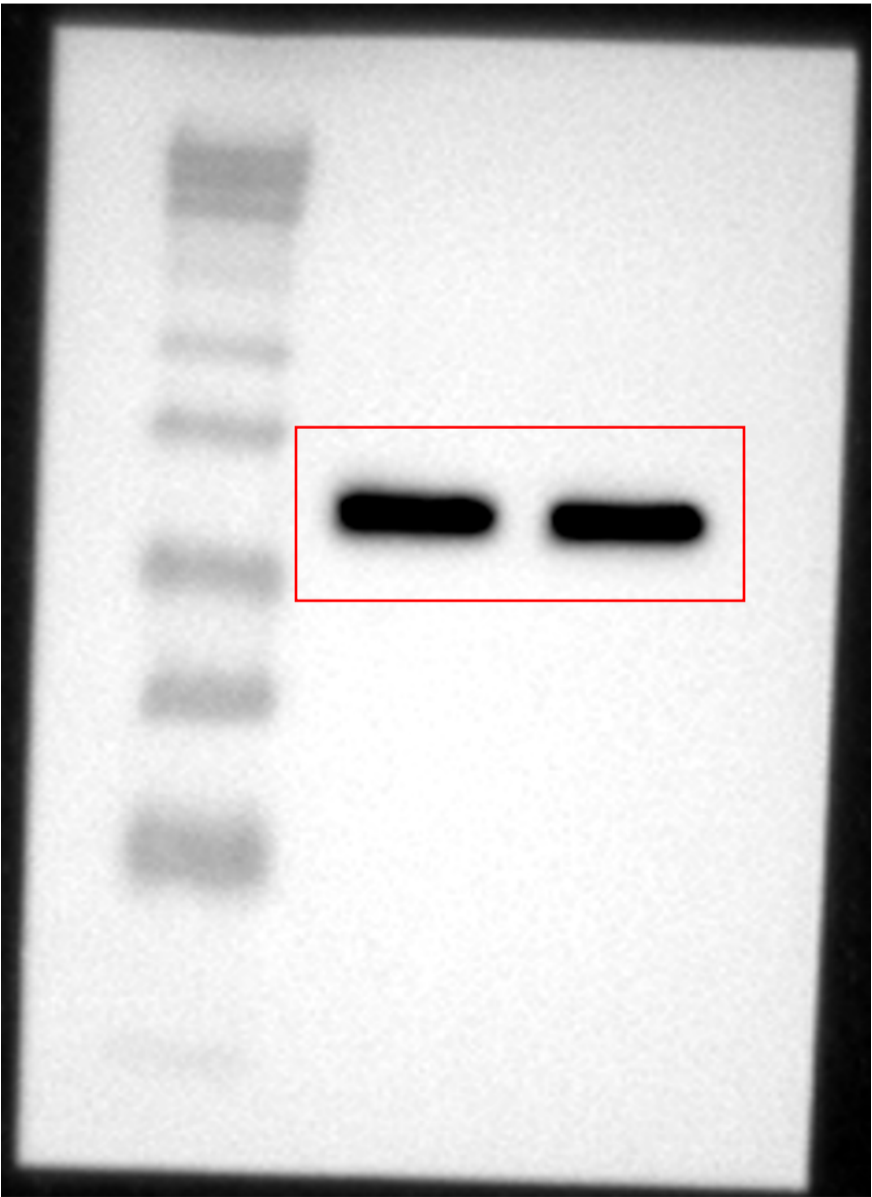

37 kDa

Supplement: S1 Raw images — (PDF) [file pone.0313939.s006.pdf]
